# Association of DNA methylation-predicted growth differentiation factor 15 with all-cause mortality among older adults in the United States: A prospective cohort study of NHANES 1999 to 2002

**DOI:** 10.1097/MD.0000000000046578

**Published:** 2025-12-12

**Authors:** Meisheng Zou, Daofan Li, Suhong Wu

**Affiliations:** a Department of Geriatrics, Zhongshan City People’s Hospital, Zhongshan City, Guangdong, China.

**Keywords:** all-cause mortality, DNA methylation, GDF15, NHANES, older adults

## Abstract

Growth differentiation factor 15 (GDF15) serves as a prominent biomarker across multiple physiological and pathological processes. Increased levels of GDF15 are associated with elevated mortality risk. Nevertheless, the prognostic utility of DNA methylation (DNAm)-predicted GDF15 concentrations for mortality remains inadequately explored. Our study utilized a national cohort to examine the link between GDF15 levels predicted by DNAm and the risk of all-cause mortality. This study employed data from the National Health and Nutrition Examination Survey (NHANES) cycles spanning 1999 to 2002. A regression model was applied to derive DNA methylation (DNAm)-predicted GDF15 concentrations. To evaluate the association linking DNA methylation-predicted GDF15 levels to mortality, we conducted adjusted Cox proportional hazards regression modeling. Dose-response relationships were evaluated using restricted cubic splines (RCS), and subgroup analyses were carried out to strengthen the findings’ robustness. Elevated levels of GDF15 predicted via DNA methylation exhibited a marked association with increased all-cause mortality risk (HR = 1.11, 95% CI = 1.05–1.18). Participants within the top tertile of epigenetically estimated GDF15 concentrations exhibited a considerably increased hazard of death (HR = 1.62, 95% CI = 1.27–2.08). Kaplan–Meier curves demonstrated gradually decreasing survival probabilities corresponding to higher epigenetically derived GDF15 levels. A nonlinear dose-response relationship between DNAm-inferred GDF15 concentrations and all-cause mortality was revealed by restricted cubic spline analysis. This positive relationship consistently maintained significance within every prespecified subgroup. Epigenetically estimated GDF15 levels represent an independent predictor of all-cause mortality. This association retains its significance in multiple analytical approaches and across various subpopulations, highlighting the potential of GDF15 as a biomarker for stratifying mortality risk. Future studies are needed to elucidate the biological mechanisms through which GDF15 operates and to evaluate its applicability in clinical settings for reducing mortality risk.

## 1. Introduction

Growth differentiation factor 15 (GDF15) is part of the Transforming growth factor β (TGF-β) superfamily of cytokines^[1]^ and has recently gained significant attention in aging research.^[2,3]^ First identified in 2010, research showed that GDF15 is associated with all-cause mortality in a cohort of Swedish men.^[4]^ High levels of GDF15 are linked to various negative health outcomes and often indicate an increased risk of mortality.^[5–7]^ GDF15 is established as a biomarker for many chronic illnesses, particularly those worsened by aging, and high levels are associated with conditions like cognitive impairment,^[8]^ diabetes,^[9]^ and heart failure.^[10]^ Nevertheless, the underlying molecular processes regulating GDF15 levels and their causal influence on mortality remain incompletely characterized.

Recent progress in epigenetic research has underscored the role of DNA methylation (DNAm) in regulating gene expression, which may affect circulating biomarker levels.^[11,12]^ Researchers are increasingly using DNA methylation as a tool to predict disease risk and mortality. Leveraging DNA methylation signatures for estimating GDF15 concentrations provides a novel approach to elucidate GDF15’s involvement in mortality and enhance prognostic models. The NHANES dataset, with its extensive data linked to the National Death Index, presents an ideal platform for examining the connection linking epigenetically derived GDF15 levels to death risk. Our research aims to assess the link between GDF15 concentrations predicted via DNA methylation and all-cause mortality.

## 2. Methods

### 2.1. Study population

**Figure 1.**
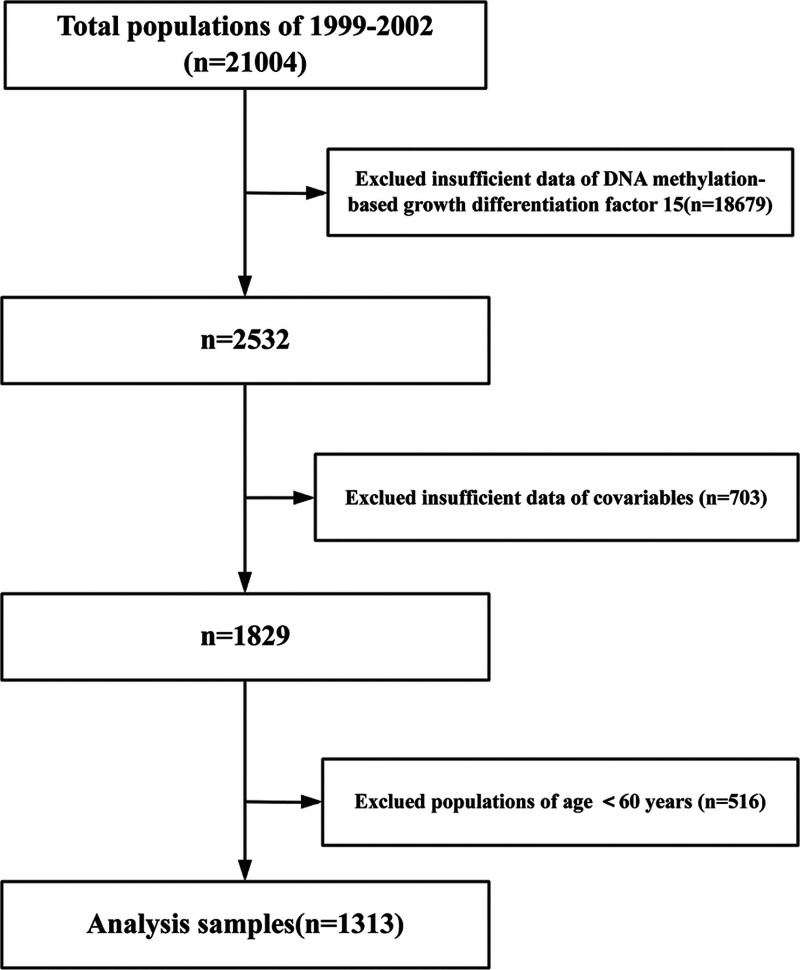
Flow chart of the study participants’ selection process.

The National Health and Nutrition Examination Survey (NHANES) database serves as the foundation for this research, having been meticulously curated via a nationwide cross-sectional study orchestrated by the Centers for Disease Control and Prevention (CDC). This initiative aims to monitor the health and nutritional status of the United States population comprehensively.^[13,14]^ Our analysis centered on information obtained from 2 discrete NHANES assessment periods (1999–2002), selected exclusively for their inclusion of complete DNA methylation profiles. The 1999-to-2002 NHANES cohort comprised 21,004 enrolled participants. Participants were excluded if they had incomplete data on DNA methylation (n = 18,679), lacked sufficient covariate information (n = 703), or were under the age of 60 (n = 516). Participants aged ≥ 60 years were included, as this threshold demarcates the onset of older adulthood and is associated with a heightened prevalence of age-related physiological changes and chronic conditions relevant to our study of DNA methylation. This focus enhances the detection of associations with age-associated epigenetic alterations. Consequently, the final analytical dataset consisted of 1313 participants (see Fig. 1). The data collection protocol was sanctioned by the National Center for Health Statistics (NCHS) Ethics Review Board (Protocols #98-12), and informed consent was obtained from all participants. Additionally, the design and reporting of this research conformed to the ethical standards set forth in the Declaration of Helsinki and adhered to the STROBE Statement guidelines.^[15]^

### 2.2. Data collection

The 1999-to-2002 NHANES surveys employed a complex, multi-phase probability-based sampling design to ensure nationally representative coverage of noninstitutionalized U.S. residents. Data collection comprised household interviews combined with MEC-based clinical assessments. Standardized questionnaires administered by certified staff captured sociodemographic characteristics, economic status, nutritional patterns, and health indicators. Clinical evaluations included anthropometric measurements (height/weight), blood pressure monitoring, and additional diagnostic procedures. Biosamples were additionally acquired for laboratory testing. Variables incorporated in our study covered: age, sex, race/ethnicity, and marital status, poverty-income ratio (PIR) and educational attainment, body mass index (BMI) and daily caloric intake, tobacco use classification (≥100 lifetime cigarettes), alcohol consumption (≥12 drinks/yr). Biochemical markers: C-reactive protein and total cholesterol (TC). Self-reported comorbidities: cardiovascular disease (CVD), chronic kidney disease (CKD), hypertension, diabetes mellitus, and malignancy history.

### 2.3. DNA methylation measurement

The 1999-to-2002 NHANES cycles enrolled adults ≥ 50 years providing blood specimens for DNA isolation. Participants represented: approximately 50% randomly sampled eligible non-Hispanic Whites, All qualifying individuals from non-Hispanic Black, Mexican American, other Hispanic, and additional racial groups. Extracted DNA from whole blood was cryopreserved at −80°C. Methylation profiling was conducted at Dr Yongmei Liu Duke University laboratory following standardized protocols: bisulfite conversion: 500 ng DNA processed with Zymo EZ Methylation kit per manufacturer guidelines. Amplification: converted DNA amplified per Illumina Infinium protocol (16 cycles: 95°C/30 s to 50°C/60 min). Hybridization: 4 μL bisulfite-treated DNA subjected to. Infinium HD Methylation workflow: overnight denaturation/amplification (20–24 hour), Fragmentation, precipitation, resuspension, EPIC BeadChip v1.0 hybridization (16–24 hour). Detection: primer extension via nucleotide labeling, imaging on Illumina iScan. For bioinformatic processing: cell composition estimated via regression calibration using DNAm data, IDOL probes + FlowSorted.Blood.EPIC_ref implemented with “estimateCellCounts2” (immunomethylomics package^[16–18]^). DNAm-predicted GDF15 levels derived from regression models adjusted for age, sex, and CpG-specific methylation values.

### 2.4. Ascertainment of mortality

Within the NHANES program, mortality information was linked to the National Death Index by the National Center for Health Statistics (NCHS) through December 31, 2019.^[19]^ Disease-specific mortality was verified using the International Statistical Classification of Diseases, 10th Revision (ICD-10).^[20]^

### 2.5. Statistical analysis

Statistical analyses incorporated NHANES-specific survey procedures to account for complex sampling design. As our cohort represented a subsample of the national survey, dedicated sampling weights were applied. Weighted cox regression models generated hazard ratios (HRs) with 95% confidence intervals (CIs) to evaluate mortality associations with DNAm-predicted GDF15. Covariate selection followed 3 criteria: univariate *P* < .1, 10% change in matched odds ratios upon inclusion, clinically relevant constraints. Model specifications: Model 1: unadjusted. Model 2: adjusted for age, sex, and ethnicity. Model 3: model 2 + education, PIR, marital status, smoking, alcohol use, caloric intake, CRP, BMI, TC, and comorbidity history (hypertension, diabetes, CVD, CKD, malignancy). Validation approaches: tertile-based categorization of DNAm-GDF15 (T1-T3), Kaplan–Meier curves comparing survival probabilities across tertiles, Restricted cubic splines examining nonlinear mortality associations.

Stratified analyses by: BMI (<25 vs ≥ 25 kg/m^2^), sex, ethnicity, PIR (<1.3, 1.3–3.5, >3.5), education (<HS, HS, >HS), smoking/alcohol status (binary), Comorbidity presence (diabetes, hypertension, CVD, CKD, malignancy). Prespecified effect modification was assessed through interaction terms with likelihood ratio testing. All computations and visualizations used R v4.3.3 (R Foundation), with statistical significance defined as *P* < .05.

## 3. Results

### 3.1. Baseline characteristics of participants

This investigation enrolled 1313 participants, averaging 70.3 ± 7.5 years in age, with males comprising 658 individuals (50.1%). Their baseline characteristics are detailed in Table 1. Compared to the lowest tertile (T1), subjects in the highest DNAm-predicted GDF15 tertile (T3) were typically older, exhibited a higher probability of being unmarried and self-identifying as Non-Hispanic White, and presented with a greater prevalence of hypertension and cancer history. Additionally, this group (T3) displayed elevated TC concentrations. Moreover, individuals categorized into T3 also reported lower average daily energy consumption and BMI values.

**Table 1:** Baseline characteristics of participants according to the tertiles of DNAm-predicted GDF15.

Variables	Total (n = 1313)	DNAm-predicted GDF15			*P* value
		T1 (n = 438)	T2 (n = 437)	T3 (n = 438)	
Gender, n (%)					0.314
Male	658 (50.1)	213 (48.6)	232 (53.1)	213 (48.6)	
Female	655 (49.9)	225 (51.4)	205 (46.9)	225 (51.4)	
Age, yr	70.3 ± 7.5	64.0 ± 3.4	70.3 ± 5.8	76.6 ± 6.6	<.001
Race, n (%)					<.001
Mexican American	377 (28.7)	154 (35.2)	138 (31.6)	85 (19.4)	
Other Hispanic	71 (5.4)	22 (5)	29 (6.6)	20 (4.6)	
Non-Hispanic White	555 (42.3)	141 (32.2)	176 (40.3)	238 (54.3)	
Non-Hispanic Black	268 (20.4)	99 (22.6)	82 (18.8)	87 (19.9)	
Other race	42 (3.2)	22 (5)	12 (2.7)	8 (1.8)	
BMI, kg/m^2^	28.5 ± 5.4	29.3 ± 5.2	28.8 ± 5.5	27.4 ± 5.4	<.001
DNAm-predicted GDF15, ng/L	1019.5 ± 143.6	873.6 ± 56.2	1008.6 ± 32.4	1176.3 ± 107.6	<.001
C-reactive protein, mg/dL	0.6 ± 1.3	0.5 ± 1.1	0.5 ± 0.8	0.7 ± 1.8	0.069
Total cholesterol, mmol/L	5.5 ± 1.0	5.6 ± 1.0	5.4 ± 1.0	5.4 ± 1.0	0.042
Daily energy intake, kcal	1715.9 ± 716.1	1796.4 ± 730.4	1732.3 ± 756.6	1619.2 ± 647.2	0.001
Marital status, n (%)					<.001
Not married	488 (37.2)	136 (31.1)	150 (34.3)	202 (46.1)	
Married or living with partner	825 (62.8)	302 (68.9)	287 (65.7)	236 (53.9)	
Education level, n (%)					0.375
Below high school level	618 (47.1)	196 (44.7)	212 (48.5)	210 (47.9)	
High school level	281 (21.4)	89 (20.3)	91 (20.8)	101 (23.1)	
Above high school level	414 (31.5)	153 (34.9)	134 (30.7)	127 (29)	
Alcohol intake (at least 12 alcohol drinks per year), n (%)					0.435
Yes	805 (61.3)	272 (62.1)	275 (62.9)	258 (58.9)	
No	508 (38.7)	166 (37.9)	162 (37.1)	180 (41.1)	
Smoking status (at least 100 cigarettes in life), n (%)					0.05
Yes	693 (52.8)	214 (48.9)	229 (52.4)	250 (57.1)	
No	620 (47.2)	224 (51.1)	208 (47.6)	188 (42.9)	
PIR, n (%)					<.001
≤1.3	413 (31.5)	125 (28.5)	143 (32.7)	145 (33.1)	
1.3–3.5	546 (41.6)	163 (37.2)	172 (39.4)	211 (48.2)	
>3.5	354 (27.0)	150 (34.2)	122 (27.9)	82 (18.7)	
Hypertension, n (%)					.013
Yes	686 (52.2)	209 (47.7)	225 (51.5)	252 (57.5)	
No	627 (47.8)	229 (52.3)	212 (48.5)	186 (42.5)	
Diabetes, n (%)					.876
Yes	248 (18.9)	82 (18.7)	80 (18.3)	86 (19.6)	
No	1065 (81.1)	356 (81.3)	357 (81.7)	352 (80.4)	
CKD, n (%)					<.001
Yes	159 (12.1)	26 (5.9)	37 (8.5)	96 (21.9)	
No	1154 (87.9)	412 (94.1)	400 (91.5)	342 (78.1)	
Cancer, n (%)					<.001
Yes	218 (16.6)	52 (11.9)	62 (14.2)	104 (23.7)	
No	1095 (83.4)	386 (88.1)	375 (85.8)	334 (76.3)	
CVD mortality, n (%)					<.001
No	1047 (79.7)	381 (87)	356 (81.5)	310 (70.8)	
Yes	266 (20.3)	57 (13)	81 (18.5)	128 (29.2)	
All-cause mortality, n (%)					<.001
No	492 (37.5)	273 (62.3)	163 (37.3)	56 (12.8)	
Yes	821 (62.5)	165 (37.7)	274 (62.7)	382 (87.2)	
Follow-up time, mo	160.3 ± 71.0	193.8 ± 56.2	167.8 ± 67.0	119.2 ± 67.8	<.001

BMI = body mass index, CKD = chronic kidney disease, CVD = cardiovascular disease, DNAm = DNA methylation, GDF15 = growth differentiation factor 15, PIR = family income to poverty ratio.

### 3.2. Epigenetically estimated GDF15 and total mortality risk in the elderly

Significant associations between DNAm-estimated GDF15 and all-cause mortality were consistently observed across all statistical models (Table 2). In the unadjusted model (model 1): continuous analysis: HR = 1.4 (95% CI = 1.35–1.45; *P* < .001) Tertile comparisons (ref = T1): T2: HR = 2.04 (1.68–2.48; *P* < .001), T3: HR = 4.67 (3.88–5.62; *P* < .001), significant dose-response gradient (Ptrend < 0.001). After demographic adjustment (model 2: age/sex/ethnicity): continuous HR attenuated to 1.15 (1.09–1.22; *P* < .001), T2: aHR = 1.28 (1.03–1.58; *P* = .023), T3: aHR = 1.91 (1.5–2.43; *P* < .001), persistent trend significance (Ptrend < 0.001). In the fully adjusted model (model 3: socioeconomic/clinical covariates): continuous HR further reduced to 1.11 (1.05–1.18; *P* < .001), T2: aHR = 1.20 (0.97–1.49; *P* = .098), T3: aHR = 1.62 (1.27–2.08; *P* < .001), maintained association gradient (Ptrend = 0.003).

**Table 2:** Hazard ratios of DNAm-predicted GDF15 in association with all-cause mortality in older adults.

All-cause mortality	Model 1		Model 2		Model 3	
	HR (95% CI)	*P* value	HR (95% CI)	*P* value	HR (95% CI)	*P* value
DNAm-predicted GDF15 as continuous variable						
Per 100 units	1.4 (1.35,1.45)	<.001	1.18 (1.09, 1.22)	<.001	1.11 (1.05, 1.18)	<.001
DNAm-predicted GDF15 as categories variable						
T1	1.0	–	1.0	–	1.0	–
T2	2.04 (1.68, 2.48)	<.001	1.28 (1.03, 1.58)	.023	1.2 (0.97,1.49)	<.001
T3	4.67 (3.88,5.62)	<.001	1.91 (1.5,2.43)	<.001	1.62 (1.27,2.08)	<.001
*P* for trend	–	<.001	–	<.001	–	.001

Model 1: not adjusted any covariates; Model 2: adjusted age, sex and ethnicity; Model 3: adjusted age, sex, ethnicity, marital status, poverty-to-income ratio, education level, smoking status, alcohol status, daily energy intake, CRP, BMI, total cholesterol, history of comorbidities (hypertension, diabetes, CVD, CKD and cancer).

BMI = body mass index, CKD = chronic kidney disease, CRP = C-reactive protein, CVD = cardiovascular disease.

**Figure 2.**
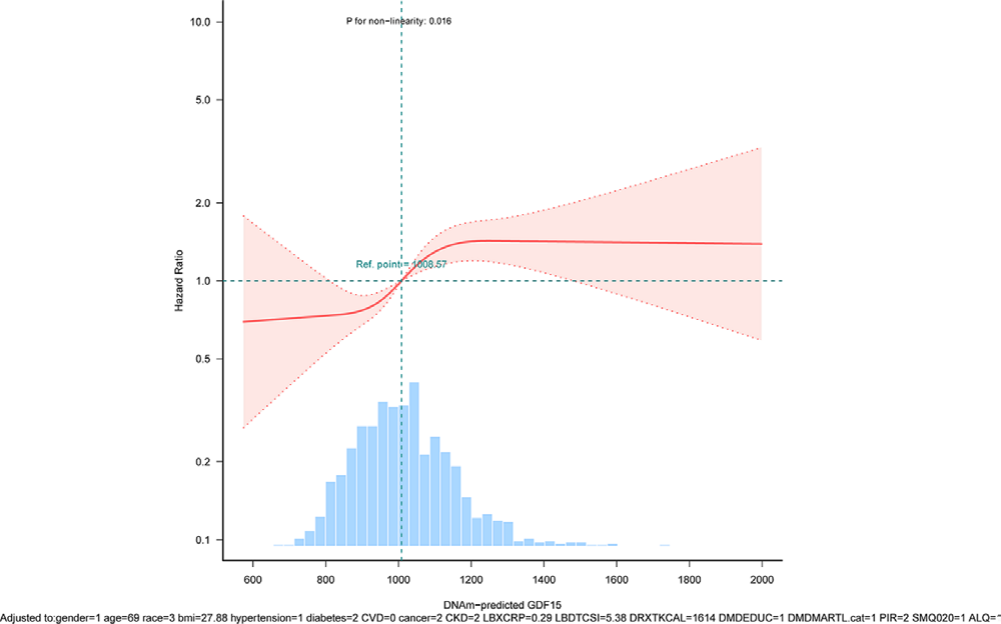
Restricted cubic spline assessment of the exposure-response relationship linking epigenetically derived GDF15 to all-cause mortality. GDF15 = Growth differentiation factor 15.

**Figure 3.**
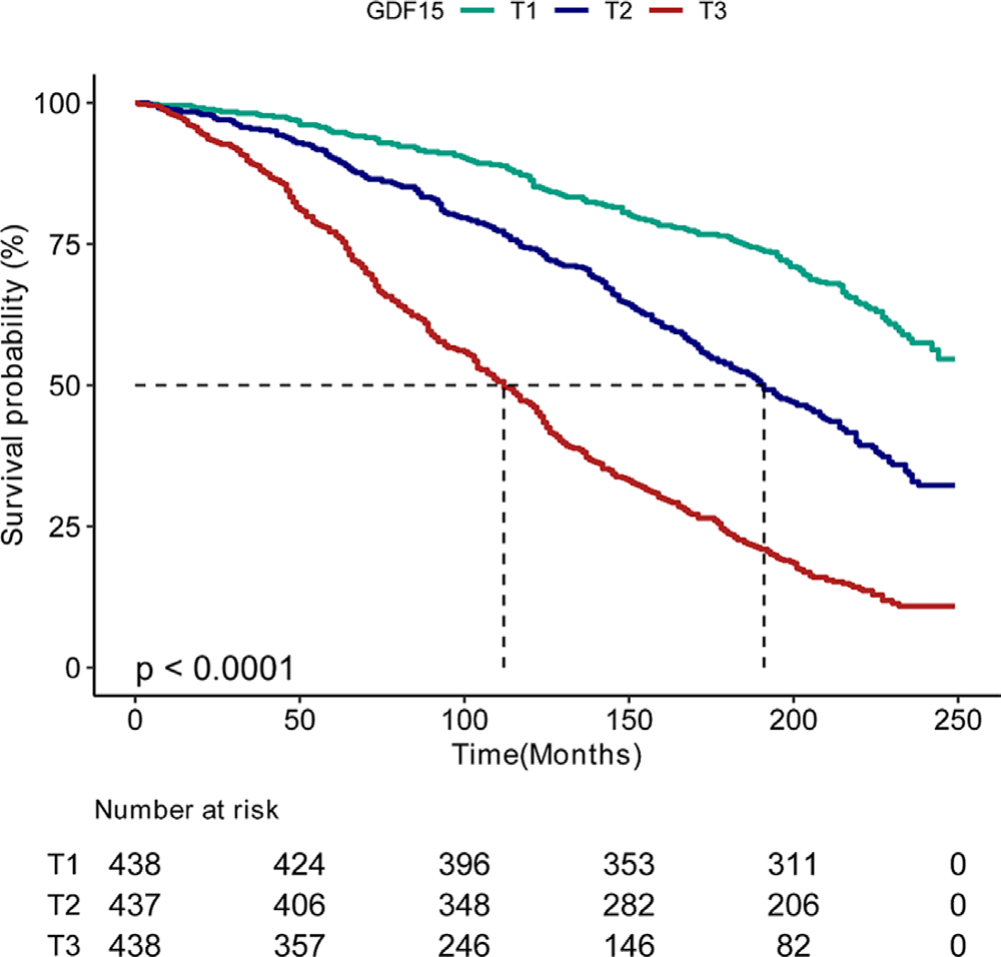
Kaplan–Meier survival curves illustrated temporal mortality patterns across GDF15 tertile strata. GDF15 = Growth differentiation factor 15.

RCS analyses revealed a nonlinear mortality association with epigenetically derived GDF15 after covariate adjustment (Fig. 2; *P* = .016). KM curves further demonstrated significantly divergent survival probabilities across GDF15 tertiles (T1–T3; *P* < .0001, Fig. 3). Relative to the reference tertile (T1), survival probabilities gradually declined with increasing GDF15 levels.

### 3.3. Subgroup analysis

**Figure 4.**
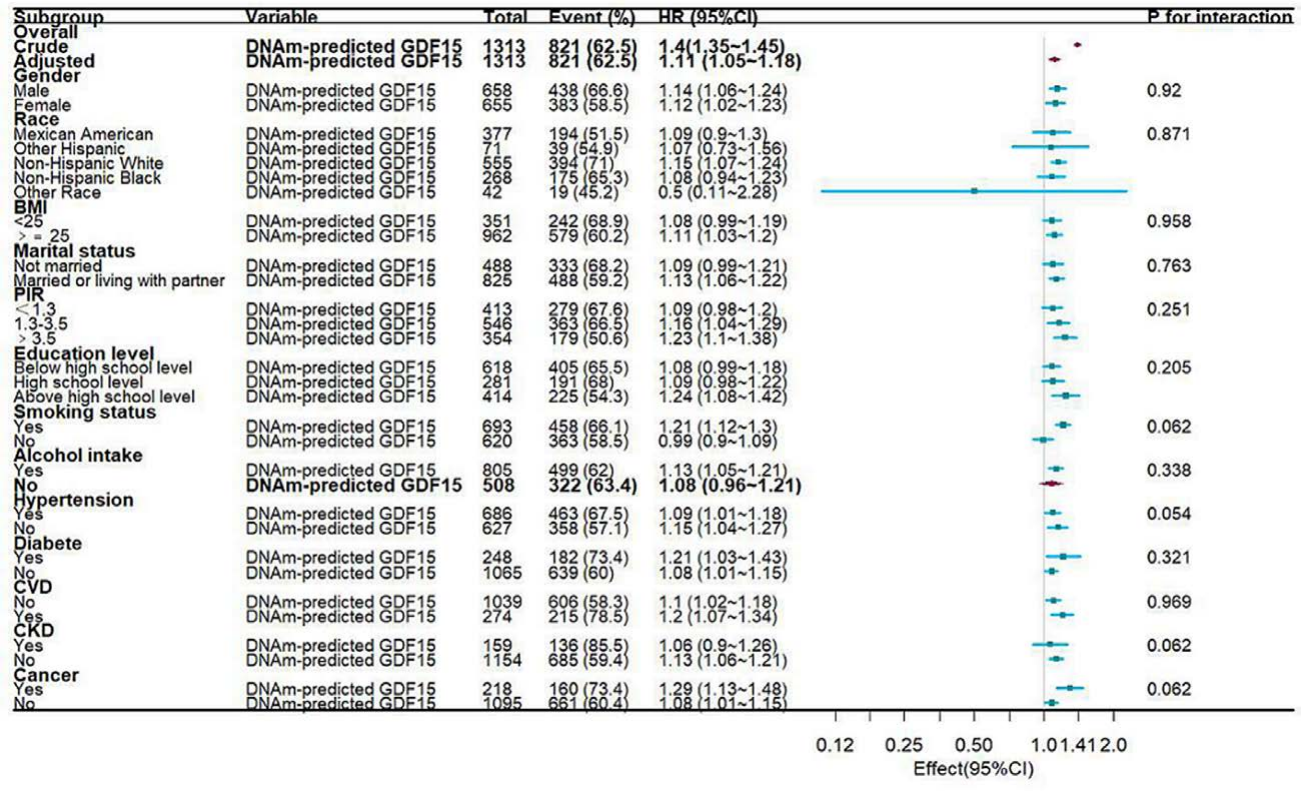
Forest plot illustrating the mortality association of epigenetically derived GDF15 (HRs with 95% CIs). CIs = confidence intervals, GDF15 = Growth differentiation factor 15, HRs = hazard ratios.

To evaluate the robustness of our findings, we performed stratified analyses across multiple demographic and clinical subgroups (Fig. 4). Stratifying participants by variables including sex, ethnicity, BMI, marital status, PIR, education level, smoking status, alcohol use, hypertension, diabetes, cardiovascular disease, chronic kidney disease, and cancer revealed no statistically significant interaction effects (all *P* for interaction > .05). Critically, the mortality association with epigenetically derived GDF15 concentrations remained consistently significant across all prespecified subgroups.

## 4. Discussion

This research addresses the critical issue of death risk in the older population by leveraging data from the NHANES 1999-to-2002 cohort. Our analysis underscores a robust association of DNAm-predicted GDF15 concentrations with mortality risk. Unlike prior investigations which often focused on single risk factors, our findings suggest the utility of GDF15 as a dependable biomarker for predicting death in this demographic. These findings suggest elevated GDF15 concentrations, derived from epigenetic signatures, may function as an early biomarker of mortality risk irrespective of demographic, behavioral, and clinical factors. This relationship was replicated across diverse analytical approaches and subgroup stratifications, supporting the need for future clinical validation studies.

Our analysis establishes a significant positive relationship linking DNAm-estimated GDF15 concentrations to overall death risk among older individuals, suggesting GDF15’s potential as a biomarker for mortality assessment in this group. This association yielded (HR) of 1.11 (95% CI = 1.05–1.18) per 100 unit increase in continuous DNAm-estimated GDF15 and 1.62 (95% CI = 1.27–2.08) for the highest tertile group. Corroborating our results, NHANES data revealed that rising DNAm-predicted GDF15 concentrations were linked to greater all-cause death risk in the general population. This association yielded HR of 1.08 (95% CI = 1.02–1.15) per unit increase in continuous GDF15 and 1.56 (95% CI = 1.16–2.10) for the highest tertile group.^[21]^ Numerous investigations employing direct blood-based measurements have established a robust association between elevated circulating GDF15 levels and all-cause mortality. For instance, research involving 1036 middle-aged subjects (n = 1036) found that elevated GDF15 levels correlated with increased death rates, especially individuals of low socioeconomic status.^[22]^ Bao et al, through a 2-decade cohort study, established GDF15 as a robust predictor of overall death risk.^[23]^ Further supporting this, GDF15 demonstrated efficacy in predicting cancer-related mortality among patients with cardiovascular risk factors.^[24]^ Collectively, these studies reinforce GDF15’s reliability as a mortality marker, particularly for older adults. Notably, findings may vary across distinct cohorts. In one investigation of 1201 heart failure patients (n = 1201), GDF15 concentrations, while associated with clinical outcomes, failed to demonstrate independent mortality prediction after multivariable adjustment.^[25]^ Binder et al similarly documented links between GDF15 concentrations and heart failure hospitalizations/mortality, though not with ventricular arrhythmias.^[26]^ These inconsistencies may stem from population diversity; cohorts with heart failure frequently present intricate comorbidity patterns that could obscure GDF15-mortality associations. Our findings demonstrate the potential of epigenetically derived GDF15 as a robust biomarker for stratifying mortality risk in older populations, necessitating further exploration of its clinical applications and mechanistic foundations.

Our findings highlight DNA methylation’s pivotal role in modulating GDF15 expression, a molecule involved in inflammatory processes and programmed cell death across diverse pathological contexts.^[27–31]^ Elevated GDF15 concentrations correlate with increased all-cause death risk, especially in older adults, suggesting that specific inflammation-associated cell subtypes may influence treatment efficacy. For example, this work shows that subjects with higher DNAm-predicted GDF15 exhibit distinct inflammatory signatures, potentially affecting their susceptibility to cardiovascular disease and cancer.^[32]^ Regulatory *T* cells, identified as another key subtype in this analysis, are crucial for sustaining immunological homeostasis and regulating inflammatory cascades.^[33]^ Subsequent research ought to delineate the primary transcription factors governing the differentiation and function of these inflammation-related cellular subsets, particularly regarding GDF15 modulation. Elucidating these pathways may reveal novel therapeutic targets for controlling inflammation and ameliorating outcomes in patients exhibiting elevated GDF15. Understanding the functional significance of these transcriptional regulators will be essential for devising approaches to mitigate inflammation’s detrimental effects and augment the efficacy of existing treatment protocols. While DNAm-predicted GDF15 reflects epigenetic regulation of the gene locus, it differs conceptually from measured circulating protein in key aspects:

Temporal resolution: DNA methylation captures stable epigenetic programming potentially preceding protein elevation, serving as an early indicator of dysregulation rather than acute secretion; tissue specificity: our blood-based epigenetic measure integrates hematopoietic cell-specific regulation, whereas circulating GDF15 represents systemic secretion primarily from parenchymal tissues during stress responses; biological interpretation: the strong association between DNAm-GDF15 and overall death risk (HR = 1.62 per SD) suggests involvement of epigenetic dysregulation in disease pathogenesis, potentially through sustained overexpression mechanisms. This contrasts with transient protein elevations reflecting acute insults. These distinctions carry important translational implications: DNAm-GDF15 may identify individuals with chronically dysregulated GDF15 pathways who could benefit from targeted epigenetic therapies, whereas serum measurements better guide acute interventions. Future studies integrating both measures are warranted to dissect directionality in this relationship.

Our study demonstrates significant findings regarding the association of DNAm-predicted GDF15 concentrations with overall death risk, assessed through multiple analytical models and subgroup stratification. However, several limitations deserve acknowledgments. Several limitations warrant consideration. First, unmeasured confounders – including genetic factors, lifestyle characteristics, or unassessed biomarkers – may have influenced mortality associations. Second, restriction to the NHANES 1999–2002 cohort introduces technical variability when integrating external datasets, potentially obscuring true effects. Finally, while our observational study demonstrated significant mortality associations with epigenetically derived GDF15, such designs fundamentally preclude definitive causal attribution. Reverse causation remains an issue, since underlying health conditions might simultaneously increase GDF15 levels and elevate death risk. Future research utilizing longitudinal designs or Mendelian randomization techniques could help address these causal concerns.

## 5. Conclusions

Collectively, our findings underscore the potential utility of DNA methylation-predicted GDF15 as a robust biomarker for assessing death risk in older adults, with no significant effect modifications detected across subgroups. These results affirm the importance of GDF15 in stratifying all-cause mortality risk, suggesting its prospective role in clinical assessment. Subsequent studies should focus on elucidating the biological pathways linking GDF15 to mortality. Ultimately, this line of inquiry may inform the creation of novel preventative strategies aimed at reducing death risk among the elderly, thereby enhancing life quality and promoting healthy aging.

## Acknowledgments

We would like to thank all NHANES participants and staff.

## Author contributions

**Formal analysis:** Meisheng Zou.

**Funding acquisition:** Meisheng Zou.

**Investigation:** Meisheng Zou.

**Resources:** Meisheng Zou.

**Software:** Meisheng Zou.

**Supervision:** Daofan Li.

**Validation:** Meisheng Zou, Suhong Wu.

**Visualization:** Meisheng Zou, Daofan Li, Suhong Wu.

**Writing – original draft:** Meisheng Zou, Suhong Wu.

**Writing – review & editing:** Suhong Wu.
